# A fragment of type VI collagen alpha-6 chain is elevated in serum from patients with atopic dermatitis, psoriasis, hidradenitis suppurativa, systemic lupus erythematosus and melanoma

**DOI:** 10.1038/s41598-023-28746-2

**Published:** 2023-02-21

**Authors:** Signe Holm Nielsen, Helena Port, Cecilie Møller Hausgaard, Jesper Grønlund Holm, Jacob P. Thyssen, Solveig Skovlund Groen, Morten Karsdal, Valdemar Wendelboe Nielsen, Alexander Egeberg, Anne-Christine Bay-Jensen, Simon Francis Thomsen

**Affiliations:** 1 Immunoscience, Nordic Bioscience, Herlev Hovedgade 205–207, 2730 Herlev, Denmark; 2 Department of Biomedical Sciences, University of Copenhagen, Copenhagen, Denmark; 3 Department of Dermatology, Bispebjerg Hospital, Copenhagen, Denmark; 4 Department of Clinical Medicine, University of Copenhagen, Copenhagen, Denmark

**Keywords:** Biochemistry, Biomarkers, Diseases

## Abstract

Extracellular matrix (ECM) remodeling of the skin is a continuous process necessary for maintaining tissue homeostasis. Type VI collagen (COL6) is characterized as a beaded filament, located in the dermal ECM, where COL6-α6-chain has been demonstrated upregulated in atopic dermatitis. The aim of this study was to develop and validate a competitive ELISA, targeting the N-terminal of COL6-α6-chain, named C6A6, and evaluate its associations with the dermatological condition’s atopic dermatitis, psoriasis, hidradenitis suppurativa, systemic lupus erythematosus, systemic sclerosis, urticaria, vitiligo, and cutaneous malignant melanoma in comparison, to healthy controls. A monoclonal antibody was raised and employed in an ELISA assay. The assay was developed, technically validated, and evaluated in two independent patient cohorts. Cohort 1 showed C6A6 was significantly elevated in patients with atopic dermatitis (*p* < 0.0001), psoriasis (*p* < 0.0001), hidradenitis suppurativa (*p* = 0.0095), systemic lupus erythematosus (*p* = 0.0032) and melanoma (*p* < 0.0001) compared to healthy donors. Cohort 2 confirmed C6A6 being upregulated in atopic dermatitis compared to healthy controls (*p* < 0.0001), but also associated with disease severity (SCORAD, *p* = 0.046) and lowered in patients receiving calcineurin inhibitors (*p* = 0.014). These findings are hypothesis generating, and the utility of the C6A6 biomarker for disease severity and treatment response needs to be validated in larger cohorts and longitudinal studies.

## Introduction

Extracellular matrix (ECM) remodeling of the skin is a continuous process necessary for maintaining tissue homeostasis. The skin can be divided into three layers; epidermis, dermis and subcutis, which all have distinct tissue architecture and function^1,2^. The dermis provides tensile strength and elasticity, in addition to being the layer where fibroblasts play a key role in ECM synthesis and maintenance of tissue structure^3,4^. Dermal ECM can be divided into the papillary and reticular dermal ECM, and is composed by matricellular proteins (COMP, SPARC, thrombospondin-1, periostin, tenascin C and X), proteoglycans (decorin, versican, biglycan, fibromodulin and lumican), collagens (I, III, V, VI, XII, XIV and XV), fibrillin microfibrils (fibrillin-1 and -2) and elastic fiber proteins (elastin, EMLIN-1 and 2, LTBP-4, fibulin-4 and -5)^2^. Dysregulation of the papillary and reticular dermal ECM remodeling is a key event in the pathology of inflammatory skin diseases, including atopic dermatitis and psoriasis^1,2,5^.

Type VI collagen is characterized as a beaded filament collagen, found in the papillary and reticular dermal ECM, where it forms a microfibrillar network^6^. Six different chains of type VI collagen (α1, α2, α3, α4, α5, α6), have been identified and are expressed across connective tissues^7^. In vitro experiments, have demonstrated how type VI collagen α1-chain is important for ECM assembly in human dermal fibroblasts, and loss of type VI collagen resulted in loss of fibroblast motility^8^. In skin pathologies, the α3-chain, measured by the serum-biomarker PRO-C6, has previously been associated with progression of systemic sclerosis^9^. Based on transcriptomics, immunohistochemical staining and mRNA analysis, the type VI collagen α6-chain gene has been described to be upregulated in patients with atopic dermatitis^10–12^. It has also been suggested that increased levels of type VI collagen in subcutaneous tissue contributes to early phases of systemic sclerosis^13^. In addition, COL6Α6 is upregulated by IL-4 and IL-13 in mRNA from human keratinocytes^10^. Generally, the importance of type VI collagen in maintenance of tissue homeostasis in the skin, has been described, and quantifying type VI collagen in patients with dermatological disorders may be a useful tool for identification of patients and development of drugs.

We hypothesized that a specific fragment of COL6Α6 could be applied as a blood-biomarker for dermatological conditions. The aim of this study was to develop and validate a competitive ELISA, targeting the N-terminal of COL6Α6, named C6A6, and evaluate its associations with the dermatological conditions atopic dermatitis, psoriasis, hidradenitis suppurativa, systemic lupus erythematosus, systemic sclerosis, urticaria, vitiligo, and cutaneous malignant melanomaand in comparison to healthy controls.

## Materials and methods

Synthetic peptides used for generation of monoclonal antibodies, assay development and assay validation were purchased from Genscript (Piscataway, NJ, US) (Table 1).

**Table 1:** Sequences of the synthetic peptides used for monoclonal antibody production, assay development and validation.

Peptide type	Sequence
Immunogenic peptide	DSGPEYADVV-GGC-KLH*
Selection peptide	DSGPEYADVV
Elongated selection peptide	QDSGPEYADVV
Truncated selection peptide	SGPEYADVV
Non-sense coating and standard peptide	YRDDLKKLLE-Biotin and YRDDLKKLLE

*Keyhole Limpet Hemocyanin.

### Monoclonal antibody development, production, and characterization

Monoclonal antibodies were generated by immunization of the following amino acid sequence ^20'^↓DSGPEYADVV ^30'^, targeting the human α6 chain of type VI collagen. Immunization was initiated by subcutaneous injection of 200 μl emulsified antigen and 100 μg of immunogenic peptide (DSGPEYADVV-GGC-KLH) in 4- to 6-week-old Balb/C mice using Stimmune (Thermo Fisher). Immunizations were repeated every second week. The mouse with the highest stable serum titer were selected for fusion and boosted intravenously with 50 μg immunogenic peptide in 100 μl 0.9% NaCl solution 3 days before isolation of the spleen for cell fusion. Hybridoma cells were produced by fusion of the mouse spleen cells with SP2/0 myeloma cells as described by Gefter et al.^14^. The generated clones were plated into a 96-well microtiter plates for further growth, and the limiting dilution method was applied to promote monoclonal growth. Reactivity to the supernatant were tested by an indirect ELISA performed on streptavidin-coated plates. DSGPEYADVV -K-Biotin was used as screening peptide, while the standard peptide DSGPEYADVV was used to further test the specificity of the newly developed antibody clones. Supernatant was collected from the hybridoma cells and purified using HiTrap affinity columns (GEHealthcare Life Science, Little Chalfront, Buckinghamshire, UK) according to manufacturer’s instructions and antibody isotype was determined using Rapid ELISA Mouse monoclonal antibody Isotyping Kit (Invitrogen, Carlsbad, CA, USA) following the manufacturer’s protocol.

Reactivity toward human serum, citrate plasma, heparin plasma, EDTA plasma and rat serum was tested with purchased samples from a commercial supplier (Valley Biomedical, Winchester, VA). The monoclonal antibodies were selected to specifically recognize the standard peptide (DSGPEYADVV), and not an elongated or truncated sequence of the peptides (QDSGPEYADVV and SGPEYADVV, respectively).

### C6A6 assay development

The development of the competitive chemiluminescence immunoassay (CLIA) included preliminary optimizing experiments to identify the right reagens, concentrations, incubation-time and -temperature. The final C6A6 competitive ELISA procedure was as follows: A 96-well streptavidin-coated white microplate (Greiner Bio-One, Kremsmünster, Austria) was coated with 3 ng/mL biotinylated synthetic peptide (DSGPEYADVV*-*K-Biotin) dissolved in assay buffer (10 mM phosphate buffered saline (PBS), 1% bovine serum albumin, 0.1% Tween-20, 0.36% Bronidox, 4 g/L NaCl, adjusted to pH 7.4 at 20 °C) and incubated for 30 min at 20 °C with constant shaking (300 rpm) in darkness. Next, 20 µL/well of standard peptide (100 ng/mL) and samples were added to the appropriate wells, followed by the addition of 100 μL/well of HRP-labelled antibody diluted in assay buffer to the concertation of 200 ng/mL and incubated for 1 h at 20 °C with constant shaking (300 rpm) in darkness. After each incubation step, wells were washed five times with the washing buffer (20 mM Tris, 50 mM NaCl, pH 7.2). The chemiluminescence substrate (Roche, BM Chemiluminescence ELISA substrate (POD), Basel, Switzerland) working solutions were mixed 15 min prior to use and 100 μL/well were added to plate and incubated for 3 min at 20 °C with constant shaking (300 rpm) in darkness. The relative light units were measured at all wavelengths within 5 min on a microplate luminometer reader (SpectraMax M5, Molecular Devices, CA, USA*)*. A standard curve was plotted using a 4-parameter logistic curve fit Y = (A − D)/(1 + (x/C)^B) + D, where R > 0.9. Data were analyzed using the SoftMax Pro version 7.0.3 software.

### Technical evaluation

Assay linearity was determined by two-fold dilutions of four human serum and two rat serum samples and calculated as percentage of recovery of the undiluted sample. To determine the assay accuracy, healthy human serum samples were spiked with standard peptide and a human serum sample with a known high C6A6 concentration and calculated as the percentage recovery of the measured value and the expected concentration of the peptide or human serum sample. Specificity of the generated monoclonal antibody was calculated as percentage of signal inhibition by two-fold diluted standard peptide (DSGPEYADVV), elongated peptide (QDSGPEYADVV), truncated peptide (SGPEYADVV), and non-sense peptide (YRDDLKKLLE). Interference of substances present in blood, was tested by adding a low/high content of hemoglobin (2.50/5 mg/mL), lipemia/lipids (1.50/5 mg/mL) and biotin (3/9 ng/mL) to a human serum sample with a known concentration of C6A6. The normal reference levels for hemoglobin, lipemia/lipids and biotin were 0–10 mg/dL (0–0.0016 mmol/L), < 150 mg/dl (< 1.69 mmol/L) and 0.22–3.00 ng/ml, respectively. The stability of the analyte was examined through temperature tests (0, 2-, 4-, 24-, and 48-h of incubation at either 4 °C or 20 °C) and five freeze–thaw cycles of human serum samples. The recovery was calculated with 0 h/0 cycle of the sample as a reference. The intra- and interassay variation was determined by 10 independent runs of five quality controls and two kit control runs in double determinations, while the measurement range was defined as the range between lower limit of measurement range (LLMR) and the upper limit of measurement range (ULMR) on the 10 runs. The standard curves from the 10 independent runs was used to determinate the IC50 (half-maximal inhibition concentration). All samples for the technical validation and biological measurements were run in double determination.

### Biological evaluation of C6A6 and patient demographics

The biological capability of C6A6 was evaluated in serum samples from two cross-sectional studies. The first cohort (Cohort 1) was obtained from the commercial vendor Proteogenex (Culver City, CA, USA), and was approved by the local ethical committee (Russian Oncological Research Center, Blokhin Rams Ethics Committee review form, Protocol no. PG-ONC 2003/1). Informed consent was obtained from all participants. The second cohort (Cohort 2) was obtained from Department of Dermatology, Bispebjerg Hospital, University of Copenhagen, Denmark. This study was approved by the Committee of Biomedical Research Ethics of the Capital Region of Denmark, where all patients filed informed consent. The healthy donor samples were acquired from BioIVT (Westerbury, NY, USA) and Lee Biosolutions (Meryland Heights, MO, USA), which obtained informed concent from all participants.

Cohort 1 included patients with atopic dermatitis (n = 20), plaque psoriasis (n = 20), hidradenitis suppurativa (n = 6), systemic lupus erythematosus (n = 12), systemic sclerosis (n = 18), urticaria (n = 19), vitiligo (n = 20) and cutaneous malignant melanoma (n = 20), and matched healthy donors (n = 24) with no symptomatic or chronic disease. Cohort 2 included patients with atopic dermatitis (n = 158) and healthy age, gender, and ethnicity matched controls (n = 22). Serum samples were collected from January 2012 and June 2018^15^. Patients with atopic dermatitis met the criteria defined by Hannifin and Rajka in 1980, assessed by a senior dermatologist. Severity was assessed by SCORAD, which is a commonly used severity index for atopic dermatitis (range 0–103 points; high score indicates severe disease). Patients were divided into disease severity groups based on SCORAD into mild (SCORAD < 25), moderate (SCORAD range 25–50), and severe disease (SCORAD > 50). Only age and gender were available for the healthy donor subjects. The study was executed in compliance with the Helsinki Declaration of 1975. Serum samples were obtained and stored at -80 °C until biomarker analysis.

### Ethical statement

All animals were treated according to the guidelines for animal welfare. Monoclonal antibody production in mice was approved by the Danish National Authority (The Animal Experiments Inspectorate) under approval number 2013–15-2934–00,956. Mice were sacrificed by cervical dislocation. The study is reported in accordance with ARRIVE guidelines.

### Statistical analysis

Patient characteristics of the two cohorts are presented as a number (frequency) and percentage for categorical variables and either mean with standard deviation or mean with range for continuous variables. Statistical differences for categorical variables were assessed using a Kruskal–Wallis test (nonparametric) for cohort 1, and Mann–Whitney U-test in cohort 2. For cohort 1, an ANCOVA analysis adjusted for age and gender was used to calculate the differences between the groups of patients. For cohort 2, an ANCOVA analysis adjusted for age was used to calculate the differences between the groups of patients ranging from 4 to 90 years of age. Graphs are shown as mean ± 95% CI. For all statistical analyses performed, a *P*-value below 0.05 was considered significant. Statistical analysis and graphs were performed using GraphPad Prism version 9 (GraphPad Software, Inc., La Jolla, CA) and MedCalc version 19.3 (MedCalc Software, Ostend, Belgium).

## Results

### Specificity, accuracy, and precision of the C6A6 assay

**Figure 1:**
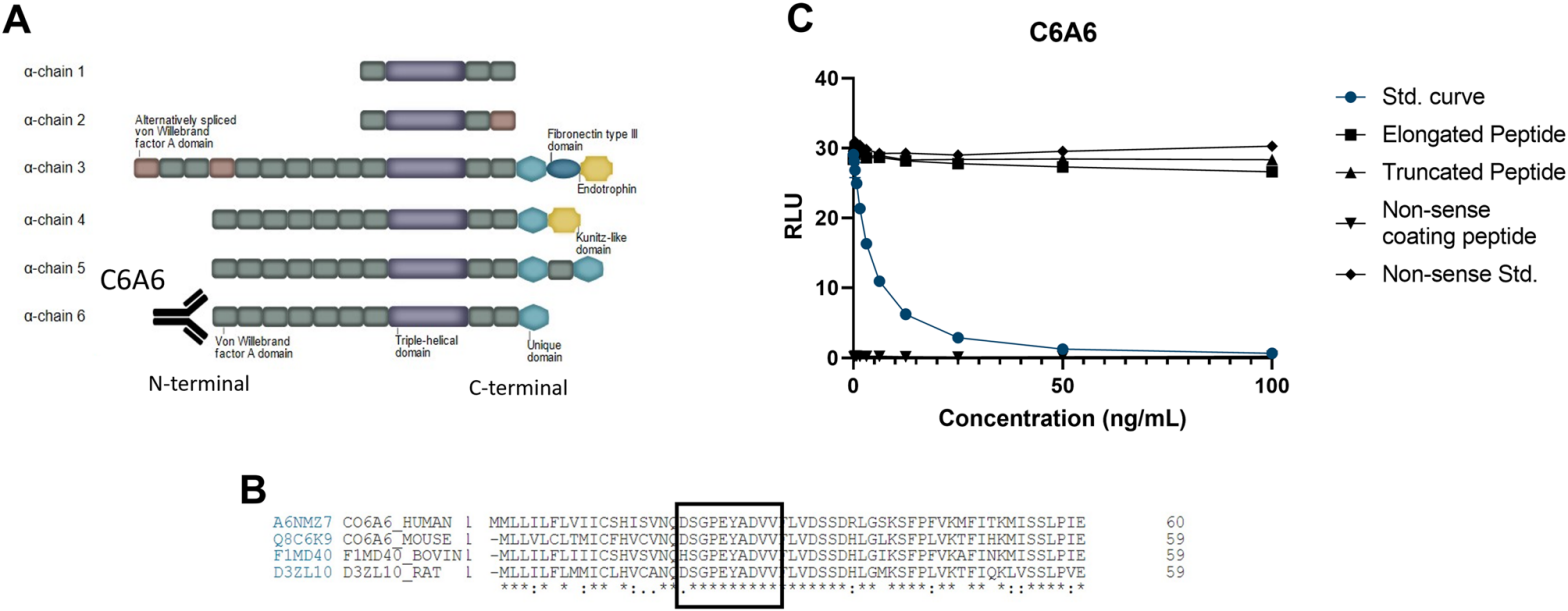
Overview of type VI collagen, the target of C6A6 and assay specificity. (**A**) The primary structure of type VI collagen including domains from all six alpha-chains, (**B**) Sequence alignment of the targeted COL6Α6 sequence in human, mouse, bovine and rat species (black box). The sequence was aligned using Uniprot, (**C**) Specificity of the C6A6 assay. Reactivity towards the standard peptide (DSGPEYADVV), truncated peptide (SGPEYADVV), elongated peptide (QDSGPEYADVV) and non-sense standard peptide (YRDDLKKLLE). No background signal was detected when coating with a non-sense coating peptide (YRDDLKKLLE-Biotin). Signals are shown as relative luminescence (RLU) per second, as a function of standard peptide.

The C6A6 assay targets the N-terminal of type VI collagen, alpha 6 chain (Fig. 1A). The human sequence was aligned using UNIPROT, and the corresponding sequence in mouse and rat are 100% aligned, while bovine has one mismatch in position 1 (Fig. 1B). The hybridomas producing the best mAbs were screened for reactivity towards the standard peptide and native material. The clone NBH-306-11 8E7-2C11-1F10-2C10 was chosen for assay development and determined as an IgG1 subtype. To evaluate the specificity of the C6A6 assay, the mAb was tested towards the elongated peptide, truncated peptide, non-sense standard peptide and non-sense coater, and showed no reactivity towards those peptides (Fig. 1C). Technical validation was performed to evaluate the novel C6A6 assay. Overall, the C6A6 assay demonstrated good performance (see Table 2), including accepted inter- and intra-variations, analytes stability and limited interference from endogenous.

**Table 2:** Summary of technical parameters for C6A6.

TECHNICAL VALIDATION	RESULTS (Accepted recovery: 100 ± 20%)
IC50	3.86 ng/mL
Measurement range (LLMR-ULMR)	0.60–13.00 ng/mL
Inter-assay variation, mean (range)	5% (7.31–18.75)
Intra-assay variation, mean (range)	12% (2.4–12.05)
Dilution recovery of human serum, mean (range)	105% (85.4–120.4)
Spiking recovery (selection peptide in serum), mean (range)	104% (104–109)
Analyte stability and recovery, 24 h at 4 °C/20 °C , mean (range)	93% (85.9–96.8)/81% (66.8–90.6)
Freeze–thaw stability, five cycles, mean (range)	97% (72.6–103.5)
Hemoglobin interference (low/high), mean (range)	102% (87.1–121.3) / 85% (76.2–98.0)
Lipemia interference (low/high), mean (range)	105% (82.6–120.5) / 107% (80.8–130.4)
Biotin interference	< 10 ng/mL

### C6A6 is elevated in patients with selected skin diseases compared to healthy controls

**Figure 2:**
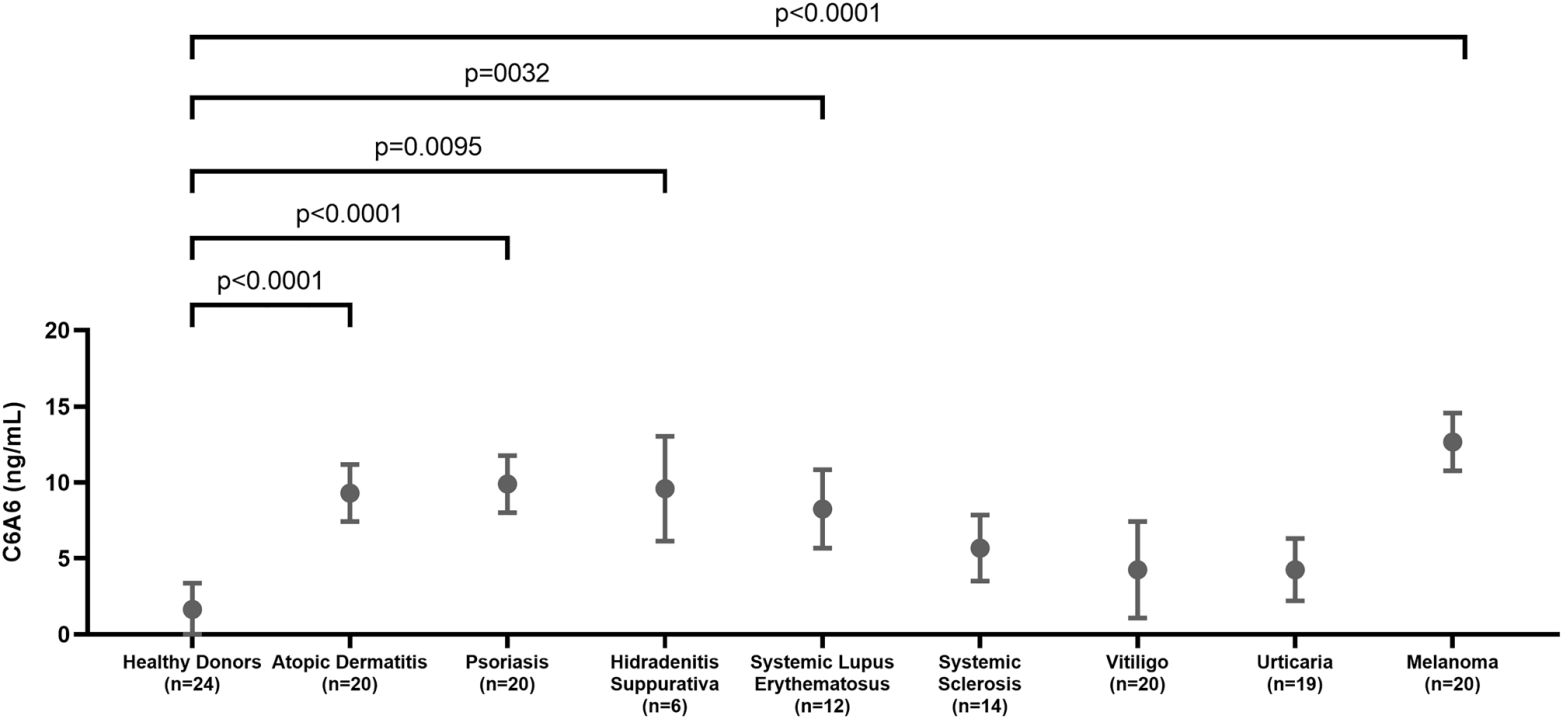
Results from cohort 1. Serum levels of C6A6 were assessed in healthy donors (n = 20), and in patients with atopic dermatitis (n = 20), psoriasis (n = 20), hidradenitis suppurativa (n = 6), systemic lupus erythematosus (n = 12), systemic sclerosis (n = 14), urticaria (n = 19), vitiligo (n = 20), and melanoma (n = 20). Mean with 95% confidence interval. Data was analyzed using an ANCOVA corrected for age and gender. Data are depicted as mean ± 95%CI.

Cohort 1 included patients with atopic dermatitis (mean age: 52.3 years, 10% male), psoriasis (mean age: 50.7 years, 10% male), hidradenitis suppurativa (mean age: 49.5 years, 0% male), systemic lupus erythematosus (mean age: 50.5 years, 16.7% male), systemic sclerosis (mean age: 54.4 years, 5.6% male), urticaria (mean age: 43.6 years, 20% male), vitiligo (mean age: 56.9 years, 25% male) and melanoma (mean age: 55.6 years, 10% male), Table 3. C6A6 was significantly elevated in serum from patients with atopic dermatitis (*p* < 0.0001), psoriasis (*p* < 0.0001), hidradenitis suppurativa (*p* = 0.0095), systemic lupus erythematosus (*p* = 0.0032) and melanoma (*p* < 0.0001) compared to healthy donors (Fig. 2). No significant difference was found between patients with systemic sclerosis, urticaria, and vitiligo and healthy controls (*p* > 0.1).

**Table 3:** Patient Demographics for Cohort 1.

	Healthy controls (N = 25)	Atopic dermatitis (N = 20)	Psoriasis (N = 20)	Hidradenitis Suppurativa (N = 6)	Systemic Lupus Erythematosus (N = 12)	Systemic sclerosis (N = 18)	Urticaria (N = 20)	Vitiligo (N = 8)	Cutaneous malignant melanoma (N = 20)	*p* value
*Sex*										
Male	11 (44.0%)	10 (50.0%)	10 (50.0%)	0 (0.0%)	2 (16.7%)	1 (5.6%)	4 (20.0%)	2 (25.0%)	10 (50.0%)	0.004
*Age*										
Mean (SD)	46.20 (11.77)	52.30 (4.11)	50.65 (6.25)	49.50 (13.66)	50.50 (5.87)	54.44 (11.91)	43.55 (16.16)	56.88 (12.97)	55.55 (9.46)	0.009
*BMI*										
Mean (SD)	NA	NA	25.71 (1.87)	NA	24.74 (2.10)	25.57 (2.68)	NA	NA	26.69 (6.33)	–
*Ethnicity*										
Black, n (%)	8 (32.0%)	0	0 (0.0%)	4 (66.7%)	0 (0.0%)	0 (0.0%)	0 (0.0%)	0 (0.0%)	0	–
Caucasian , n (%)	17 (68.0%)	20	20 (100.0%)	0 (0.0%)	12 (100.0%)	4 (22.2%)	0 (0.0%)	0 (0.0%)	20 (100%)	
Non-Hispanic/Latino, n (%)	0 (0.0%)	− 100%	0 (0.0%)	0 (0.0%)	0 (0.0%)	10 (55.6%)	16 (80.0%)	7 (87.5%)	0	
Unknown, n (%)	0 (0.0%)	0	0 (0.0%)	2 (33.3%)	0 (0.0%)	4 (22.2%)	4 (20.0%)	1 (12.5%)	0	

Categorical variables are written as number (percentage), while continuous variables are mean (standard deviation). BMI, body mass index.

### C6A6 is associated with disease severity in atopic dermatitis and lower in patients treated with topical immunomodulatory treatment

**Figure 3:**
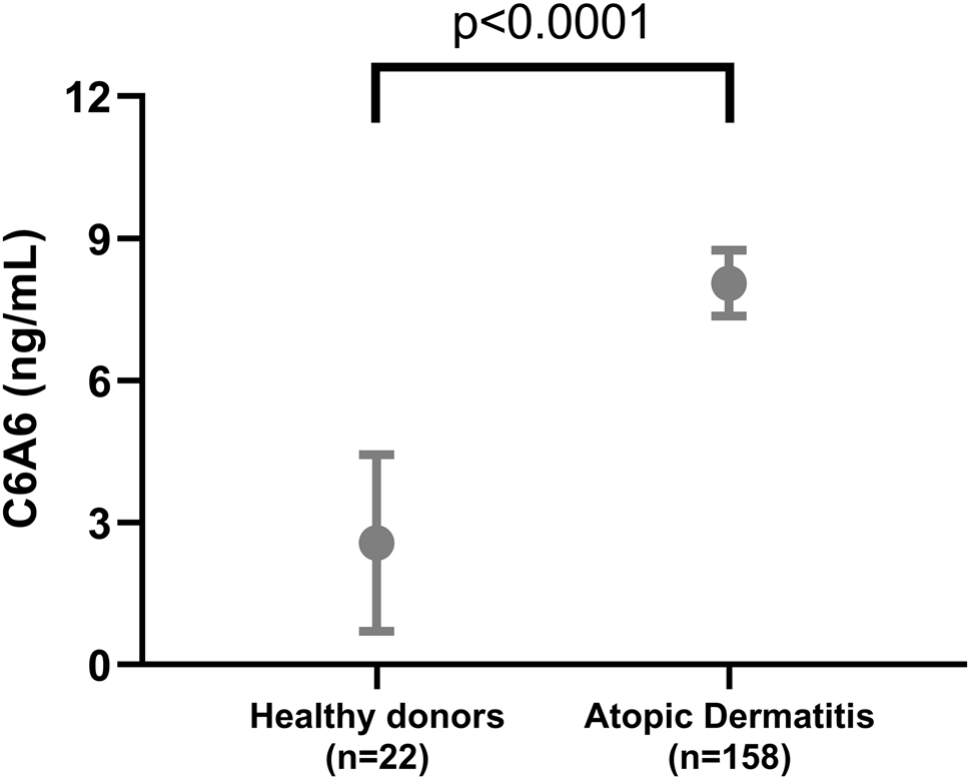
Levels of C6A6 in healthy donors (n = 22) and patients with atopic dermatitis (n = 158). C6A6 levels in healthy donors and patients with atopic dermatitis. Mean with 95% confidence interval. Data was analyzed using an ANCOVA corrected for age. Data are depicted as mean ± 95%CI.

**Figure 4:**
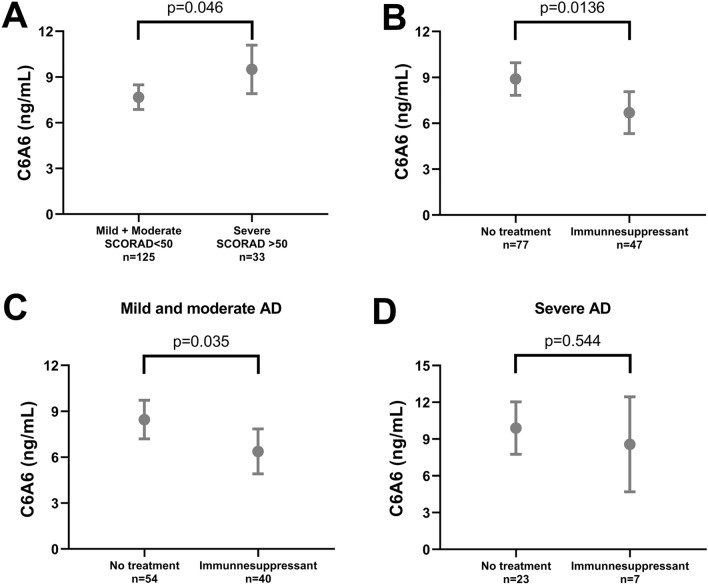
Levels of C6A6 stratified into disease severity and treatment. (**A**) Levels of C6A6 divided into mild and moderate AD (SCORAD; 0–50), and severe AD (SCORAD; < 50), (**B**) Levels of C6A6 divided into AD patients’ immunosuppressant treatment (topical calcineurin inhibitors), (**C**) Levels of C6A6 for mild and moderate AD patients receiving immunosuppressant treatment, (**D**) Levels of C6A6 for severe AD patients receiving immunosuppressant treatment. Mean with 95% confidence interval. Data was analyzed using an ANCOVA corrected for age. Data are depicted as mean ± 95%CI.

Cohort 2 included 158 patients with atopic dermatitis (mean age: 30.0, 52% male), and 22 healthy donors (mean age: 29.6, 50% male), Table 4. Patients with atopic dermatitis had significantly higher levels of C6A6 compared to healthy controls (*p* < 0.0001, Fig. 3). Out of 158 atopic dermatitis patients, 53 (33.3%) had mild disease, 72 (45.3%) had moderate disease and 33 (21.4%) had severe disease. We evaluated whether the C6A6 assay was associated with disease severity, by comparing patients with severe atopic dermatitis (SCORAD > 50) to patients with mild and moderate atopic dermatitis (SCORAD 0–50). C6A6 was significantly higher in patients with severe atopic dermatitis, compared to mild and moderate atopic dermatitis (*p* = 0.046, Fig. 4A). Out of 158 patients, 47 patients received topical immunomodulatory treatment with calcineurin inhibitors pimecrolimus or tacrolimus. Patients receiving treatment, had significantly lower levels than patients not receiving treatment (*p* = 0.014, Fig. 4B). In addition, patients with mild and moderate atopic dermatitis receiving topical calcineurin inhibitors had significantly lower levels of C6A6, compared to those without treatment (*p* = 0.035, Fig. 4C), while there was no difference in severe atopic dermatitis patients receiving topical calcineurin inhibitors (*p* > 0.1, Fig. 4D).

**Table 4:** Patient Demographics for Cohort 2.

	Healthy donors (n = 22)	Atopic dermatitis (n = 158)	*p*
Males, n (%)	11 (50%)	82 (52%)	0.827
Age at sampling, mean (range)	29.6 (6.0–65.0)	30.0 (4.3–90.9)	0.902
*SCORAD, n *			
Mild (< 25) Moderate (25–50) Severe (> 50)		53 (33.5%) 72 (45.6%) 33 (20.9%)	
Itch (VAS)		5.7 (5.6)	
Sleep Impact (VAS)		3.1 (3.2)	
FLG-mutation (R2447X, R501X and 2282del4)		33 (20.9%)	
Asthma		74 (46.8%)	
Allergic rhinoconjunctivitis		89 (56.3%)	
Urticaria		64 (40.5%)	
Food Allergy		84 (53.2%)	
Eosinophil count (× 109/L)		0.43 (0.43)	
Serum total IgE (103 IU/L)		1,282.3 (2,088.8)	
*Systemic treatments *			
Cyclosporine, n (%) Methotrexate, n (%) Prednisolone, n (%) Azathioprine, n (%) Number of pts on systemic treatment, n (%)		2 (1.2%) 2 (1.2%) 29 (18.4%) 14 (8.9%) 37 (23.4%)	

Categorical variables are written as number (percentage), while continuous variables are mean (standard deviation). SCORAD, SCORing Atopic Dermatitis; FLG, Filaggrin gene; VAS, visual analogue scale; pts, Patients.

## Discussion

In this study, we developed and characterized a competitive ELISA for detection of C6A6 using a monoclonal antibody targeting the N-terminal of type VI collagen alpha-6 chain. The main findings of this study were as follows: 1) development of a robust and specific C6A6 assay towards the sequence DSGPEYADVV; 2) C6A6 was detectable in human, mouse and rat serum; 3) C6A6 was upregulated in patients diagnosed with atopic dermatitis, melanoma, psoriasis, hidradenitis suppurativa and systemic lupus erythematosus, compared to healthy donors; 5) C6A6 was associated with severe atopic dermatitis and decreased with immunomodulatory treatment. To our knowledge, this is the first study to show that C6A6 can be measured in serum from patients with dermatological conditions and is associated with disease severity and lowered in patients treated with topical immunomodulators in atopic dermatitis.

The C6A6 assay is characterized as a technically robust and accurate assay by showing acceptable dilution recovery, interference, and stability tests. The intra- and inter-variation was accepted with values of 5% and 12%, respectively. The assay was further characterized as being specific towards the N-terminal site of C6A6 after cleavage of the signal peptide.

Type VI collagen is located in the papillary and reticular dermal ECM and collagen VI microfibrils are closely associated with the basement membrane in skin, blood vessels and muscle, kidney and nerves, where they serve to attach the basement membrane into the surrounding extracellular matrix^16^. Type VI collagen is produced by fibroblasts^1–3^. Fibroblasts are activated and produce collagens in different disorders, including fibrosis and cancer. In dermatological diseases, fibroblasts are known to be implicated in impaired wound healing , fibrosis and cause extensive tissue remodeling^4^. As an example, in hidradenitis suppurativa fibroblasts are known to contribute to tunnel formation and inflammation, and thereby contribute to disease progression^1^, while in atopic dermatitis, different subpopulations of fibroblasts have been identified from lesions, where especially the inflammatory fibroblast population is suspected to signal to immune cells to mediate ECM breakdown, fibrotic remodeling and T-cell recruitment^2^.

Previously, the type VI collagen alpha-6 chain has been found to be upregulated in the blood vessels in the fibrotic dermis of wild type mice^17^ and in patients with atopic dermatitis by other analysis methods: transcriptomics, immunohistochemical staining and mRNA analysis^10–12^. These findings suggest that this particular collagen chain may be biologically relevant for patients with atopic dermatitis, and a blood-based biomarker may therefore be a tool to detect changes of this protein in circulation. Based on mRNA analysis from human keratinocytes, the level of type VI collagen alpha-6 was suppressed by addition of IL-4 and IL-13^10^. In this study, we found C6A6 to be suppressed in patients with mild and moderate atopic dermatitis when treated with topical calcineurin inhibitors. Topical calcineurin inhibitors, including pimecrolimus and tacrolimus, are widely used as first-line immunosuppressant topical treatment for atopic dermatitis and also in psoriasis^18^. This generates the hypothesis that C6A6 may be associated with treatment response. On the contrary, we did not see any inhibition in severe atopic dermatitis patients, which may indicate this group of patients need another type of treatment than topical calcineurin inhibitors. Nevertheless, this finding may be due to statistical power, with only seven patients in the treatment group. Topical calcineurin inhibitors are use for treatment of atopic dermatitis and can be found in two forms–tacrolimus (0.03–0.1% oinment, used for mild-moderate atopic dermatitis) or pimecrolimus (1.0%, used for moderate-severe atopic dermatitis) cream^19^. The drugs inhibit synthesis of proinflammatory cytokines and are used as an alternative to corticosteroids. Specifically, immunosuppressive activity results from suppressing the calcineurin activity, where calcineurin inhibitors have anti-inflammatory activity due to T-helper activity affected synthesis and release of pro-inflammatory cytokines. Tacrolimus and pimecrolimus inhibits mast cell and neutrophil activation, while releasing inflammatory mediators. Tacrolimus affects the basophils and eosinophil function, as well as apoptosis of Langerhans cells^20,21^.

Limitations of the study include assessment of C6A6 in relatively small cohorts with a cross-sectional design. Moreover, only limited clinical information was available from the two investigated patient cohorts. In conclusion, the C6A6 assay showed a high specificity towards the N-terminal peptide and was elevated in patients with dermatological conditions. It was able to distinguish AD patients from healthy donors, demonstrating a high discriminative power. The C6A6 assay was also upregulated in patients with severe AD, indicating that severe patients experience more fibroblast activity and general tissue remodeling. This may also be the case for the other skin indications, and not a specific AD remodeling process. In addition, we found the biomarker levels to be lowered in patients treated with topical immunomodulators (calcineurin inhibitors), indicating this biomarker may be associated with treatment response. These findings are hypothesis generating, and the utility of the C6A6 biomarker for disease severity and treatment response needs to be validated in larger cohorts and longitudinal studies.

## Data Availability

All data from this study are included in this article. Data are kept on file and are not publicly available due to privacy data legislation. Requests can be directed to the corresponding author.
